# Population genetics of purple saxifrage (*Saxifraga oppositifolia*) in the high Arctic archipelago of Svalbard

**DOI:** 10.1093/aobpla/plt024

**Published:** 2013-04-08

**Authors:** Maria Pietiläinen, Helena Korpelainen

**Affiliations:** Department of Agricultural Sciences, University of Helsinki, PO Box 27 (Latokartanonkaari 5), Helsinki FI-00014, Finland

**Keywords:** Arctic, ITS sequencing, microsatellites, population genetic structure, *Saxifraga oppositifolia*

## Abstract

We investigated patterns of genetic variability in *Saxifraga oppositifolia* in the isolated Arctic Svalbard archipelago. The genetic analysis included genotyping using nine polymorphic microsatellite markers and sequencing of the nuclear internal transcribed spacer region. Among populations, mean allele numbers per microsatellite locus ranged from 2.0 to 2.6, and 9 % of alleles were unique. Observed (*H*_O_) and expected (*H*_E_) heterozygosities averaged 0.522 and 0.445, respectively. Typically negative but non-significant *F*_IS_ values (mean −0.173) were found in *S. oppositifolia* populations. *F*_ST_ values were relatively low (mean 0.123). The Bayesian structure analysis provided additional information on population genetic structures. Seven out of 11 studied populations, including populations located both near each other and far apart (distances 5–210 km), showed relatively homogeneous clustering patterns, while one population located on a slope in the main settlement of Longyearbyen possessed a unique genetic structure. The Mantel test proved that there is no significant correlation between genetic and geographical distances. Different growth habits (compact, trailing and intermediate) did not possess distinct genetic compositions based on microsatellite variation. Internal transcribed spacer sequencing revealed 12 polymorphic sites. Among 24 sequenced Svalbard samples, eight haplotypes were detected, none shared by the mainland samples. Population genetic structures of *S. oppositifolia* in Svalbard show that both genetic variation and differentiation levels are modest, outcrossing is the main mating system, and dispersal and gene flow are important, probably attributable to strong winds and human and animal vectors.

## Abstract (precis)

Using DNA markers and sequencing we investigated patterns of genetic variability in purple saxifrage, *Saxifraga oppositifolia* in the isolated Arctic Svalbard archipelago. Purple saxifrage is a circumpolar, ecologically and morphologically variable species with a wide range of habitats. Population genetic structures showed that both genetic variation and differentiation levels are modest, outcrossing is the main mating system, and dispersal and gene flow are important, likely accountable to strong winds and human and animal vectors. Different growth habits (compact, trailing and intermediate) did not possess distinct genetic composition.

## Introduction

In natural environments, organisms are typically exposed to several stress factors simultaneously, and the stresses are often most pronounced in extreme habitats, such as those at high latitudes and altitudes. For instance, Arctic plants may encounter many unique environmental factors, such as drought, permafrost, nutrient leaching, cryoturbation, extreme temperatures and short growing seasons (Körner 2003). The extent to which an organism is able to deal with stress determines the limits of its ecological amplitude. It is well established that biological systems are dynamic: genetic variation enables adaptation through selection, while in small and isolated populations random evolutionary processes, such as genetic drift, may become strong. The consequences of evolutionary actions will be visible in the pattern of genetic diversity and differentiation of populations.

Purple saxifrage*, Saxifraga oppositifolia,* is an Arctic–Alpine early flowering perennial herb. It is a circumpolar, ecologically and morphologically variable species with a wide range of habitats. It probably has the widest global distribution in the family Saxifragaceae (Webb and Gornall 1989). *Saxifraga oppositifolia* grows in dry to moist soil and from sea level up to 4500 m in the Alps, including the coldest known places with angiosperm plant life (Körner 2011). It is also mentioned as the northernmost vascular plant species, having been found up in northern Greenland at 83°15′ (Gjaervoll and Rönning 1999). *Saxifraga oppositifolia* is not endangered at the moment, but climate change, the potential warming or drying of northern areas, and increased UV radiation could become a threat in the future (Körner 2003). It is self-compatible but mainly outcrossing and depends on its pollinators, which are mainly bumblebees (*Bombus* sp.) (Stenström and Molau 1992; Stenström and Bergman 1998). However, there are no bumblebees in some Arctic regions, such as Svalbard, where the pollinating insects are probably mainly small insects of the order Diptera (Coulson *et al.* 2003).

Previous population genetic and phylogeographic analyses on *S. oppositifolia* include random amplification of polymorphic DNA studies by Gabrielsen *et al.* (1997) and Gugerli *et al.* (1999), amplified fragment length polymorphism (AFLP) studies by Alsos *et al.* (2007), Kropf *et al.* (2008), Müller *et al.* (2012) and Winkler *et al*. (2012), restriction fragment length polymorphism investigations (Abbott *et al.* 1995; Abbott and Comes 2004) and studies based on cpDNA and nuclear internal transcribed spacer (ITS) sequences (Holderegger and Abbott 2003; Winkler *et al.* 2012). All these DNA studies have used universal binary markers or sequence data. On the other hand, our study utilizes microsatellite markers that we recently developed for *S. oppositifolia* (Pietiläinen and Korpelainen 2010), which, besides allowing precise genetic analyses, make it possible to draw conclusions on the effects of mating systems on population genetic structures.

In this study we aimed to reveal patterns of genetic variability in *S. oppositifolia* in the isolated Arctic Svalbard archipelago. We hypothesized that (i) populations possess low levels of genetic variation and are genetically differentiated due to the colonization history and small population sizes and consequent effects of genetic drift, (ii) inbreeding and heterozygote deficiency are negligible due to supposedly prevalent outcrossing, and (iii) founder dynamics combined with gene flow from various sources has generated an admixed genetic structure in populations exposed to considerable human interference when compared with pristine populations. Since *S. oppositifolia* is morphologically variable and presents different growth habits (compact, trailing and intermediate; Aiken *et al.* 2005), even within populations, we also investigated whether such morphological features have any relationship with the pattern of genetic variability. In addition, we compared nuclear ITS sequence variation of plants from Svalbard and more southern mainland regions of distribution in order to reveal relationships among plants across a wider geographic area.

## Methods

### Sampling, morphological observations and DNA analyses

**Figure 1.**
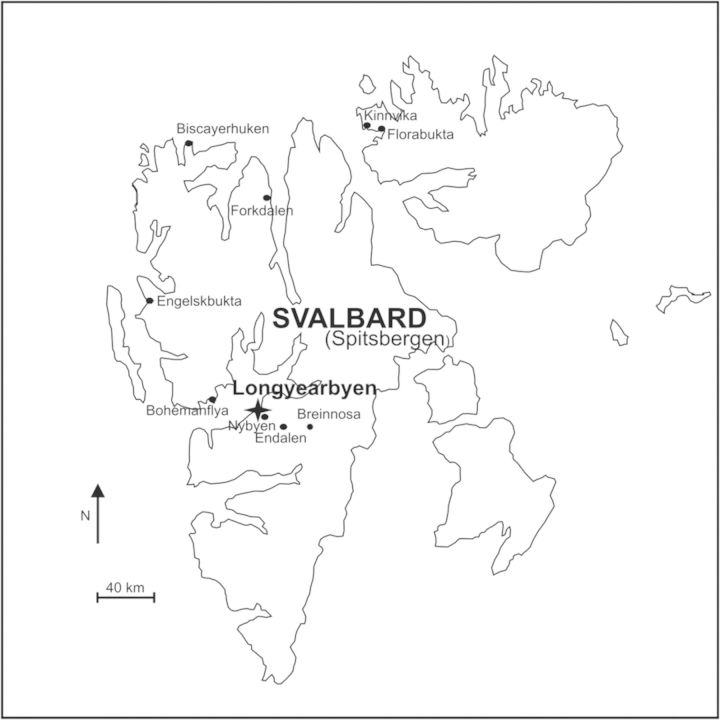
The study area of *S. oppositifolia* in Svalbard. Sampling details are given in Table 1.

We sampled fresh leaf material from 11 populations in late June 2005 (except population NYB in late June 2010) in the Arctic Svalbard archipelago in locations between latitudes 78 and 80°N (Table 1; Fig. 1). The growth form of the sampled plants was classified as compact, trailing (creeping) or intermediate. Pairwise distances between populations varied from 5 to 210 km. The collected leaf samples were desiccated in silica gel and stored at −80 °C until DNA extraction, which was conducted using commercial kits (DNeasy Plant Mini Kit, Qiagen Inc., and E.Z.N.A. Plant DNA Miniprep Kit, Omega Bio-tek, Inc.) following the manufacturers' instructions.

**Table 1:** Sampling sites, coordinates and numbers of samples collected from each *S. oppositifolia* population in Svalbard, studied using microsatellite markers (MS) and ITS sequencing (ITS).

Population	Locality	Latitude	Longitude	Sample size	
				MS	ITS
BIS	Biscayerhuken	N79°50′18.9″	E12°23′30.1″	20	3
FOR	Forkdalen	N79°31′49.8″	E15°12′33.3″	14	2
ENG	Engelskbukta 1	N78°51′16.8″	E11°43′36.7″	24	2
ED	Engelskbukta 2	N78°51′16.0″	E11°43′42.0″	17	1
BOH	Bohemanflya	N78°24′22.6″	E14°41′25.7″	29	1
LYR	Longyearbyen	N78°14′46.0″	E15°27′56.0″	28	5
NYB	Nybyen	N78°12′05.1″	E15°35′26.9″	22	0
END	Endalen	N78°11′12.2″	E15°43′07.1″	20	1
BRE	Breinnosa	N78°11′01.5″	E16°43′23.4″	21	3
KIN	Kinnvika	N80°03′15.2″	E18°15′10.5″	22	5
FLO	Florabukta	N80°01′48.5″	E18°41′33.7″	14	1

We determined the population genetic characteristics of the populations using the following nine polymorphic microsatellite markers developed for *S. oppositifolia* (Pietiläinen and Korpelainen 2010): SaxJC, SaxS1C, SaxS2C, SaxT9C, SaxT10C, S1_2, S4_2, S8_2 and S21_2. In genotyping, one of the primers in each primer pair was fluorescently (FAM, HEX or TET) labelled. Amplifications were performed in 5-µL reactions, containing 0.5 µL of DNA (about 5 ng), 2.75 µL of ddH_2_O, 0.5 µL of 10× buffer, 0.1 µL of dNTP mix (10 mM), 0.15 µL of DyNAzyme II DNA polymerase (Finnzymes) (2 U µL^−1^) and 0.5 µl of both primers (5 pmol µL^−1^). The polymerase chain reactions (PCR) were carried out as follows: DNA denaturation at 94 °C for 4 min followed by 30 cycles of denaturation at 94 °C for 45 s, annealing at 46–60 °C (depending on the primer pair) for 45 s, and elongation at 72 °C for 1 min, with a final elongation at 72 °C for 10 min. For genotyping, PCR products were first diluted with ddH_2_O. Following this, 2 µL of each sample were pipetted into a 96-well plate, and 8 µL of standard (MegaBACE ET400-R, GE Healthcare) diluted with ddH_2_O (1/45) were added. The plates were then run with the MegaBACE 1000 DNA Analysis System (Amersham Biosciences). Genotyping results were then analysed with the MegaBACE Fragment Profiler 1.2 (Amersham Biosciences).

To complement microsatellite analyses, we sequenced 24 samples from Svalbard (Table 1) and five samples from mainland Norway for the nuclear ITS region using primers ITS1 (5′-TCC GTA GGT GAA CCT GCG G-3′) and ITS4 (5′-TCC TCC GCT TAT TGA TAT GC-3′) (White *et al*. 1990). Sample sizes of populations LYR and KIN were larger (5) because LYR was of special interest due to the expected great anthropogenic influence, while KIN was an opposite example. The amplified region covered the whole distance from the end of the 18S rRNA gene to the beginning of the 26S rRNA gene. Polymerase chain reaction amplifications were performed in a total volume of 20 μL containing 2 μL of DNA (about 20 ng), 13 μL of ddH_2_O, 2 μL of 10× buffer, 0.4 μL of dNTP mix (10 mM), 0.6 μL of Dynazyme II DNA polymerase (2 U μL^−1^) and 1 μL of both primers (5 pmol μL^−1^). The PCR cycle was similar to that in microsatellite genotyping, but the annealing temperature was 50 °C. Amplification products were run on a 1 % agarose gel, and the DNA fragments were excised and purified prior to sequencing using the E.Z.N.A. Gel Extraction Kit (Omega Bio-Tek). Purified DNA samples were sequenced at Macrogen Inc. using PCR primers. Internal transcribed spacer sequences were aligned with CLUSTAL W (Thompson *et al.* 1994), and any variable sites were recorded.

### Data analyses

Based on microsatellite data, we calculated mean numbers of alleles per locus, mean observed and expected heterozygosities (*H*_O_ and *H*_E_), *F*-statistics estimators *F*_ST_ and inbreeding coefficients *F*_IS_, and conducted Hardy–Weinberg equilibrium (HWE) testing for all loci with χ^2^ tests, and performed a Mantel test to discover possible relationships between genetic and geographical distances, all using Arlequin v3.5.1.2 (Excoffier and Lischer 2010). The Bonferroni correction was applied for the tests. In addition, we performed a Bayesian analysis of population structure using the software Structure 2.3 (Pritchard *et al.* 2000) to determine the number and the distribution of genetic clusters among all samples. The correlated allele frequency model was used, which assumes that at each locus allele frequencies are correlated. With this assumption, the model infers a population structure with *K* number of clusters based on the genotype data. An admixture model was applied. At each stage, the analysis was repeated with different values of *K* (range 1–15) to discover the value with the highest estimate of log-likelihood probability (ln Pr(*X*|*K*)) of the data. For each value of *K*, 10 independent runs were conducted with a burn-in length of 100 000 iterations, followed by a data collection period of 10^6^ iterations. Furthermore, to identify the correct number of clusters (*K*) that best explains the data, Δ*K* values were calculated according to Evanno *et al.* (2005).

Since vegetative propagation is a potential mode of reproduction in *S. oppositifolia*, we used the Excel Microsatellite Toolkit (http://www.animalgenomics.ucd.ie/sdepark/ms-toolkit/) in each population to distinguish plants with a common clonal ancestry from plants sharing the same microsatellite-based multilocus genotype (MLG) by chance. When MLGs were found more than once in a population, the software MLGsim (Stenberg *et al.* 2003) was used to calculate the *P*_SEX_ values (probability that two individuals would share the same MLG by chance) and to simulate the corresponding critical values for the significance level *P* < 0.05. To obtain the critical values for the test statistic, *P*_SEX_, the number of simulations was increased until stable critical values were reached.

We constructed haplotype networks for the ITS sequence data, including all our own data and comparable sequences of the same species available in GenBank, using the network building software TCS 1.2.1 (Clement *et al.* 2000), which uses statistical parsimony (95 %) and the genealogical reconstruction algorithms of Templeton *et al.* (1992). Indels were treated as a fifth state and coded as a single mutational event.

## Results

Identical MLGs were found in all populations, except in BIS and FLO. The total percentage of MLGs was 21 % of all genotyped individuals. In most cases the number of plants sharing the same MLG was two. However, according to the simulated critical values of *P*_SEX_, obtained using the program MLGsim for each population with MLGs, none of the MLGs were likely to represent clones (*P* > 0.05). Therefore, no further adjustments due to MLGs were made for the data set.

Among populations, mean allele numbers per locus were relatively even, ranging from 2.0 (NYB and FLO) to 2.6 (BOH) (Table 2). Only 3 out of 32 alleles (9 %) were unique, i.e. found in only one population. Two of them were present in LYR and one in ENG. Observed (*H*_O_) and expected (*H*_E_) heterozygosities varied between 0.367–0.695 and 0.375–0.525, and averaged 0.522 and 0.445, respectively. Hardy–Weinberg equilibrium testing conducted for populations indicated significant heterozygote deficiencies and excesses at 5 and 23 % of loci, respectively. Inbreeding coefficients (*F*_IS_) were commonly negative (mean −0.173), indicating slight heterozygote excesses, but the values were statistically non-significant in all populations.

**Table 2:** Within-population genetic diversity in *S. oppositifolia* populations in Svalbard based on nine microsatellite markers. *A*, mean number of alleles per locus; *H*_O_, observed heterozygosity over loci; *H*_E_, expected heterozygosity over loci; *F*_IS_, fixation index over loci.

Population	*A*	*H*_O_	*H*_E_	*F*_IS_
BIS	2.4	0.375	0.450	0.167
FOR	2.4	0.575	0.509	−0.130
ENG	2.2	0.499	0.423	−0.180
ED	2.2	0.534	0.458	−0.166
BOH	2.6	0.488	0.391	−0.248
LYR	2.3	0.460	0.375	−0.227
NYB	2.0	0.550	0.403	−0.365
END	2.1	0.566	0.435	−0.301
BRE	2.1	0.632	0.515	−0.227
KIN	2.3	0.695	0.525	−0.324
FLO	2.0	0.367	0.413	0.111
Mean	2.2 ± 0.2	0.522 ± 0.099	0.445 ± 0.052	−0.173

**Figure 2.**
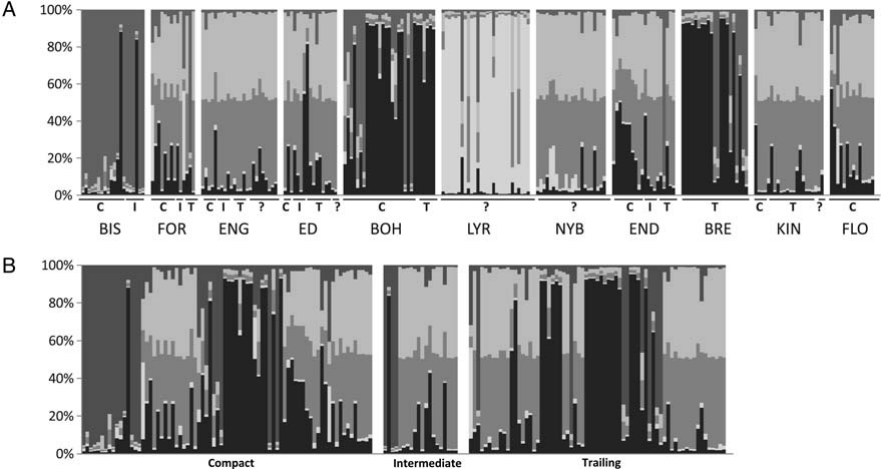
Assignment of individual samples of *S. oppositifolia* to five different gene pools as inferred by Bayesian clustering analysis. Results shown (A) for all 11 populations and (B) separately for plants with compact (C), intermediate (I) or trailing (T) growth habit. Population details are given in Table 1 and Fig. 1. The question mark (?) means that growth habit was not recorded.

The mean *F*_ST_ among all populations equalled 0.123, showing significant differentiation (*P* < 0.001). The Mantel test revealed that there is no significant association between genetic and geographic distances. Population structures were also assessed using the Bayesian structure analysis by determining the log-likelihood probability of the data (ln Pr(*X*|*K*)) as well as the rate of change values (Δ*K*). The highest Δ*K* value was detected at *K* = 5 (Fig. 2a). Seven populations (FOR, ENG, ED, NYB, END, KIN and FLO) showed relatively similar clustering patterns and their pairwise *F*_ST_ values ranged from 0 to 0.083. Populations BOH, BRE and also BIS resembled each other (pairwise *F*_ST_ equalling 0.024–0.070), while LYR possessed a more unique clustering pattern (pairwise *F*_ST_ compared with all other populations varying between 0.287 and 0.383). No evident genetic difference was detected between different growth habits (compact, trailing and intermediate) within populations (Fig. 2a) or across populations (Fig. 2b).

**Figure 3.**
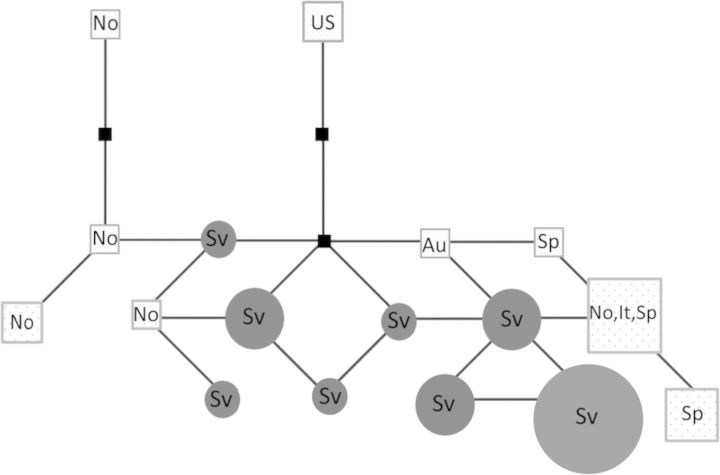
Haplotype network showing the relationships among 24 ITS sequences from Svalbard (Sv), five ITS sequences from mainland Norway (No) produced in this study and a total of 11 ITS sequences from mainland Norway (No), Austria (Au), Italy (It), Spain (Sp) and the USA (US) available in GenBank. The sizes of the circles and squares are proportional to the numbers of sequences with the same haplotype. Small squares on connecting spans indicate minimum numbers of individual mutations.

The ITS region was sequenced for 24 samples from Svalbard (GenBank accession numbers EF687783–EF687806) and five samples from mainland Norway (accession numbers EF687807–EF687811). The clear, recorded region included most of the ITS1 region, complete 5.8S rRNA and most of ITS2. Additionally, 11 ITS sequences previously available for *S. oppositifolia* in GenBank (accessions AF502089, AF504544, AY354296–AY354303 and AY357945) were included in the network analysis. In all, nine single-nucleotide polymorphisms and three 1-bp indels were detected. The 24 Svalbard sequences resulted in eight haplotypes, among which one haplotype with 11 samples was dominant (Fig. 3). The 14 sequences from mainland Europe included nine haplotypes, and the two sequences from the USA possessed a similar unique haplotype. Svalbard samples and mainland samples possessed no shared haplotypes. The five sequenced LYR samples resulted in four different haplotypes, two representing the dominant Svalbard haplotype but three sequences being unique.

## Discussion

Genetic characteristics of species impact their capacity to maintain their populations and colonize new areas. The presence of genetic diversity is especially important for plant populations in highly stochastic environments like the Arctic. The Arctic climate has been warming and the surface air temperature has increased, especially in the Atlantic region around the Svalbard archipelago (Przybylak 2007). More new areas for colonization are made bare by the retreating glaciers, and early successional species like *S. oppositifolia* may benefit from this. Müller *et al.* (2012) have actually shown that *S. oppositifolia* is a plant that can rapidly colonize new deglaciated areas without losing genetic diversity. In addition, Alsos *et al.* (2007) have suggested that past recurrent glacial cycles during the geological history have probably selected for a highly mobile Arctic flora in places like the Svalbard archipelago. The high levels of genetic diversity found in several species previously studied in Svalbard are consistent with the view that multiple long-distance dispersals have occurred (Alsos *et al.* 2007).

In the present study, although 21 % of the genotyped samples possessed MLGs that were identical to another MLG in the same population, none of the MLGs were likely to represent clones (*P* > 0.05) based on the testing of the *P*_SEX_ values obtained. However, potential smaller clones were excluded on the grounds of the random sampling strategy. Among populations, mean allele numbers per locus ranged from 2.0 to 2.6, and only 3 out of 32 alleles (9 %) were unique. Observed (*H*_O_) and expected (*H*_E_) heterozygosities averaged 0.522 and 0.445, respectively. The expected heterozygosities and allele numbers indicate a modest and relatively even level of genetic variation. In a previous AFLP-based study on *S. oppositifolia* in the Alps and Pyrenees and Sierra Nevada, Kropf *et al.* (2008) discovered *H*_E_ values varying regionally between 0.110 and 0.163, but such low values may be attributable to the different types of markers used. Also based on AFLP markers, Müller *et al.* (2012) found genetic diversity values varying between 0.19 and 0.29 in glacier foreland populations in Svalbard. However, variability comparisons on *S. oppositifolia* between the present and previous studies are difficult, as the present study is the only one utilizing microsatellites.

Typically negative but non-significant *F*_IS_ values (mean −0.173) found in *S. oppositifolia* populations indicate that outcrossing is the main mating system despite the species being self-compatible and dependent on its pollinators. At individual loci, significant heterozygote deficiencies were rare (5 %) but excesses were more common (23 %). The typical pollinators of *Saxifraga*, bumblebees (*Bombus* sp.), do not occur in Svalbard, where it needs to rely on other available pollinators, such as insects of the order Diptera (Coulson *et al.* 2003). Previously, besides the absence of bumblebees, geographic isolation by mountains and fjords as well as strong winds have also been hypothesized to lead to reduced cross-pollination (Stenström and Bergman 1998). However, the population genetic structures we detected do not indicate that crosspollination would be rare.

Relatively low *F*_ST_ values (mean 0.123) indicated a fair level of gene flow between populations occurring in different parts of Svalbard. Relatively low levels of differentiation have also been found in *S. oppositifolia* by Kropf *et al.* (2008), who in an AFLP-based study reported Nei's genetic distances of 0.022–0.030 between regions in mountain areas in Central and Southern Europe. Previous genetic work on other *Saxifraga* species utilizing AFLP markers has revealed high levels of differentiation. *Saxifraga callosa* was studied in the Alps and showed extensive differentiation (*G*_ST_ = 0.899) (Grassi *et al.* 2009), which indicated limited gene flow among refugial areas. *Saxifraga cernua* and *S. sibirica* were investigated with AFLP markers in the Ural Mountains and were found to possess considerable levels of differentiation, *F*_ST_ values equalling 0.452 and 0.362, respectively (Kapralov *et al.* 2006).

Besides *F*_ST_-based differentiation measurements, the Bayesian structure analysis provided additional information on the population genetic structures. Seven out of 11 studied *S. oppositifolia* populations (FOR, ENG, ED, NYB, END, KIN and FLO), including populations located both near each other and far apart (distance variation 5–210 km), showed relatively homogeneous clustering patterns. Another set of three populations (BIS, BOH and BRE, pairwise geographic distances 52–206 km) resembled each other, while population LYR, located on a slope next to a new housing area and building sites in the main settlement of Longyearbyen, possessed a unique genetic structure. It is likely that the human-mediated colonization history and dispersal have been extensive, and that is reflected in the present genetic composition of *S. oppositifolia* in Longyearbyen. As expected from the Bayesian structure results, the Mantel test results proved that there is no significant correlation between genetic and geographical distances.

Different growth habits (compact, trailing and intermediate) of *S. oppositifolia* did not possess distinct genetic compositions based on microsatellite variation. For instance, population BOH containing mainly compact individuals and population BRE containing only trailing plants were genetically quite similar (*F*_ST_ = 0.070). Yet, earlier research on growth and reproduction patterns in *S. oppositifolia* has indicated that morphological variability is adaptive (Brysting *et al.* 1996; Kume *et al.* 1999). As the present work is based on the use of putatively neutral microsatellite markers, genetic differentiation at adaptive loci remains unobserved if not linked with the studied loci.

Sequencing of the nuclear ITS region revealed 12 polymorphic sites. Among 24 sequenced Svalbard samples, a total of eight haplotypes were detected, none shared by the mainland samples. One major Svalbard haplotype (46 % of samples) was found in seven populations (BIS, FOR, BOH, LYR, BRE, KIN and FLO), which are located at distances 9–198 km from each other. Among the five samples sequenced for LYR, four haplotypes were found, two of which were unique among the study material. Based on the network analysis conducted, one of the unique ITS sequences of LYR appeared to descend from a haplotype discovered among samples from mainland Norway. These observations also support the view of the distinct colonization history of the LYR population. Among the nine mainland haplotypes, the Norwegian ones were related to each other or Svalbard haplotypes, except for a sample from Hordaland in southern Norway, which was identical to a haplotype also found in mountainous areas of Italy and Spain. Otherwise, the haplotypes from Austria, Italy and Spain were relatively closely related, while the haplotype revealed for the two samples from the USA was distinct. An increased sample size in mainland Europe, especially in Norway, could have provided a more precise insight into the relationship between Svalbard and other populations. Recently Winkler *et al*. (2012) discovered in their plastid sequence study that the haplotype of the single Svalbard sample of *S. oppositifolia* was also found in mainland Norway, Greenland, Slovakia and Switzerland.

In a related species, *Saxifraga paniculata*, Reisch (2008) discovered that Arctic populations contained less AFLP variation and fewer chloroplast haplotypes than populations from other regions of the distribution. The present study on *S. oppositifolia* indicates a fair level of haplotype diversity in the Arctic Svalbard populations. The ITS region as such is not supposed to be especially useful when studying larger-scale phylogeography of *S. oppositifolia* because of its largely unresolved topology with low statistical support (Holderegger and Abbott 2003). Yet, the moderate variability of the ITS region detected in our study, eight haplotypes just within Svalbard and nine additional haplotypes among the small set of samples from mainland Europe, provides interesting implications for phylogeographic studies on different geographic scales. For comparison, Winkler *et al*. (2012) discovered 27 haplotypes in a global study on 114 *S. oppositifolia* individuals based on plastid sequence data (psbA–trnH and trnTF intragenic spacers).

Strong winds in the open landscape, sea currents, drift ice and dispersal by vectors, such as humans and birds, may explain the present phylogeographic and population genetic patterns of *S. oppositifolia*. Small seeds are dispersed by the winds across the glaciers and whole plants have been known to float great distances on ice (Hultén and Fries 1986; Holderegger and Abbott 2003). It has also been reported that local reindeer forage on *S. oppositifolia*, particularly on its nutritious reproductive shoots (Cooper and Wookey 2003), especially in the northern parts of the archipelago where moss and lichen are scarce (Staaland and Punsvik 1980). Although Svalbard reindeer tend to remain in the local home range area, individual animals migrate regularly and plant dispersal may be more common than expected (Hansen *et al.* 2009). Previously, Alsos *et al.* (2007) proved by AFLP fingerprinting that long-distance colonization of Svalbard by *S. oppositifolia* has occurred repeatedly and from several source regions, probably by wind and drifting sea ice. Evidently, establishment limits distribution more than dispersal.

## Conclusions

Population genetic structures detected in *S. oppositifolia* in Svalbard revealed that differentiation levels are modest, and that dispersal and gene flow are important phenomena within Svalbard, probably attributable to strong winds and human and animal vectors. The nine polymorphic microsatellite markers used and ITS sequencing are useful tools when examining population genetics and phylogeography of *S. oppositifolia* on different geographic scales.

## Sources of Funding

Financial support was obtained from Kuopion Luonnon Ystäväin Yhdistys Foundation and NordForsk.

## Contributions by the Authors

M.P. collected most samples, conducted all laboratory work and part of the data analysis and manuscript preparation. H.K. collected some samples and conducted part of the data analysis and manuscript preparation.

## Conflicts of Interest Statement

None declared.
